# Machine Learning Algorithms for Predicting the Recurrence of Stage IV Colorectal Cancer After Tumor Resection

**DOI:** 10.1038/s41598-020-59115-y

**Published:** 2020-02-13

**Authors:** Yucan Xu, Lingsha Ju, Jianhua Tong, Cheng-Mao Zhou, Jian-Jun Yang

**Affiliations:** Department of Anesthesiology, Pain and Perioperative Medicine, The First Affiliated Hospital of Zhengzhou University, Henan, China

**Keywords:** Surgical oncology, Risk factors

## Abstract

The aim of this study is to explore the feasibility of using machine learning (ML) technology to predict postoperative recurrence risk among stage IV colorectal cancer patients. Four basic ML algorithms were used for prediction—logistic regression, decision tree, GradientBoosting and lightGBM. The research samples were randomly divided into a training group and a testing group at a ratio of 8:2. 999 patients with stage 4 colorectal cancer were included in this study. In the training group, the GradientBoosting model’s AUC value was the highest, at 0.881. The Logistic model’s AUC value was the lowest, at 0.734. The GradientBoosting model had the highest F1_score (0.912). In the test group, the AUC Logistic model had the lowest AUC value (0.692). The GradientBoosting model’s AUC value was 0.734, which can still predict cancer progress. However, the gbm model had the highest AUC value (0.761), and the gbm model had the highest F1_score (0.974). The GradientBoosting model and the gbm model performed better than the other two algorithms. The weight matrix diagram of the GradientBoosting algorithm shows that chemotherapy, age, LogCEA, CEA and anesthesia time were the five most influential risk factors for tumor recurrence. The four machine learning algorithms can each predict the risk of tumor recurrence in patients with stage IV colorectal cancer after surgery. Among them, GradientBoosting and gbm performed best. Moreover, the GradientBoosting weight matrix shows that the five most influential variables accounting for postoperative tumor recurrence are chemotherapy, age, LogCEA, CEA and anesthesia time.

## Introduction

Colorectal cancer is a common malignant tumor with high morbidity and mortality in clinical practice. It ranks third in mortality among all tumors^1^. Approximately 1.4 million new cases are diagnosed every year, and about half of the new cases are in the progressive stage. The 5-year survival rate is 30% ~ 40%, due primarily to postoperative recurrence and metastasis, of which 10% ~ 30% have abdominal cavity metastasis, with a median survival of 7 months. In China, the incidence and mortality of colorectal cancer rank third and fifth, respectively, among systemic tumors. Currently, the main clinical approach is surgical treatment assisted with multi-disciplinary methods such as radiotherapy, chemotherapy and targeted therapy. However, a meta-analysis of 18 clinical trials shows that patients have a recurrence rate of 80.00% within 3 years after surgery^2^.

With early diagnosis and treatment, the prognosis of early stage colorectal cancer patients is optimistic, and the middle and long-term survival rate is usually high. However, as early symptoms are not typical, they are easily ignored by patients, leading to progression to the middle and late stages when they are finally admitted to hospitals. This inhibits treatment and reduces long-term survival rates.

Recent machine learning (ML) methods have shown accurate predictive ability, and have been increasingly used in the diagnosis and prognosis of various diseases and health conditions^3,4^. The ML approach is a data-driven analysis method that integrates multiple risk factors into a predictive algorithm^5^. Over the past several decades, ML tools have become increasingly popular with medical researchers. Various ML algorithms, including decision tree^6^ and support vector machine (SVM)^7^, have been applied to detect key features of patients’ conditions and to model disease progression after treatment with complex health information and medical datasets. Meanwhile, studies^8^ have shown that ML models can be constructed with sex, age and complete blood cell count data to detect early colorectal cancer. They are also more suitable than non-metastatic models for predicting the survival of non-metastatic colorectal cancer.

Therefore, this study was conducted to explore whether ML algorithms can predict postoperative cancer progression in patients with stage IV colorectal cancer.

## Materials and Methods

### Patients and features

This research is a secondary analysis of data from the BioStudies (public) database (https://www.ebi.ac.uk/biostudies/studies/S-EPMC6054421). According to BioStudies’s instructions, these data have been approved by the author and can be provided to interested researchers around the world. Therefore, using the database in research does not need the approval of a secondary ethics committee. Thus, our institutional review committee also waived the requirement of written informed consent.

Patients with stage IV colorectal adenocarcinoma who had undergone primary and metastatic tumor resection surgery between January 1, 2005 and December 31, 2014 were selected from the hospital’s electronic medical database. Patients lacking demographic and pathological details or postoperative analgesia data were excluded. A total of 999 patients with stage IV colorectal adenocarcinoma were included in the training data and test data. Information such as demographic characteristics, pre-treatment CEA levels, pathologic features, and whether preoperative or postoperative adjuvant chemotherapy or radiation therapy had been used was collected. The current status of each patient was determined by follow-up recordings in outpatient clinic or subsequent admission information. The radiologists and colorectal surgeons in the hospital determined whether cancer was progressing. This was primarily based on imaging studies (e.g., CT, magnetic resonance imaging, bone scans), and defined by the Response Evaluation Criteria in Solid Tumors (RECIST) guidelines. The date of death was determined by medical records or death certificates.

Data were extracted by professional anesthesiologists who did not participate in the data analysis. The quality of the extracted data was verified by random sampling, and the data were collected up through August 2016. The primary endpoint was progression.

Patient demographic and baseline characteristics were presented with descriptive statistical methods. Continuous variables were described with mean and standard deviation (SD), or median and quartile ranges, while categorical variables were described with counts and percentages. For continuous variables with normal or asymmetric distributions, a Student’s t-test or Mann-Whitney U-test was used, respectively, to test for differences in tumor recurrence between the groups. The research samples were randomly divided into training group and testing group at a ratio of 8:2. The multiple interpolation method was adopted to supplement missing variable values.

### ML algorithms

In the present study, four basic ML algorithms—logical regression^9^, decision tree^10^, GradientBoosting^11^ and lightGBM^4,12^—are implemented^13,14^. Logistic regression is a classical classification method in statistical learning. It can be divided into binomial logistic regression and multinomial logistic regression. Decision tree is an ML method for solving classification problems. It consists of a root node, several internal nodes and several leaf nodes. The leaf nodes correspond to decision results, and each of the other nodes corresponds to a feature test. The sample set contained in each node can be divided into child nodes according to the feature values, and the root node contains the full set of samples. The path from the root node to each leaf node corresponds to a decision test sequence.

Boosting is an ML technique that can be used for regression and classification problems. It produces a weak prediction model (such as decision tree) at each step, and weights it into a total model. If weak model prediction at each step generates unanimous gradient direction of loss function, then it is called gradient Boosting.

LightGBM is a distributed gradient elevation framework based on decision tree algorithms. LightGBM applies the histogram algorithm, which has low internal storage and low data separation complexity. LightGBM uses a leaf-wise growth strategy to identify the leaf with the largest split gain (generally the largest amount of data) from all current leaves, and then splits the cycle. However, it grows a deeper decision tree, resulting in overfitting. Therefore, LightGBM adds a maximum depth limit above leaf-wise to prevent over-fitting while ensuring high efficiency.

### Hyperparameter initialization and optimization

ML algorithms involve many hyperparameters that need to be prepared before running them. In contrast to the parameters learned through training, the hyperparameters determine the structure of the ML algorithm and how to train it. The initial value of the hyperparameters for each ML algorithm used in this study was the default value specified in the package based on recommendations or experience^15^. For detailed parameterization of the algorithms, please refer to the scikit-learn user manual at http://scikit-learn.org/stable/supervised_learning.html^16^.

Performance index accuracy, sensitivity, specificity and area under receiver operating characteristic (ROC) curve are used to evaluate machine learning algorithm performance. The ROC curve shows the algorithm tradeoff setting for different thresholds for the predicted posterior probability. Precision: The proportion of positive data predicted correctly over total positive data predicted. Recall rate: the proportion of data predicted as positive cases over actual positive cases. The accuracy formula is defined as the ratio of the number of samples correctly classified by the classifier over the total number of samples for a given test data set:

$$F1=\frac{2}{\frac{1}{P}+\frac{1}{R}}=\frac{2\,\ast \,P\,\ast \,R}{P+R}$$

Mean Square Error (MSE):

$${\rm{MSE}}=\frac{1}{n}\mathop{\sum }\limits_{i=1}^{n}{({\hat{{\rm{y}}}}_{i}-{y}_{i})}^{2}$$

### Software

Descriptive and inferential statistical analysis was conducted with R. The machine learning algorithm was applied with Python 3.6 using the SCIKIT-LEARN 0.19.1 software package (SCIKIT-LEARN, http://scikit-learn.org/) (Python Software Foundation, HTTPS://www.python.org/).

## Results

999 patients who met the inclusion criteria were included in this study, of which there were 778 patients in the relapse group and 221 patients who did not relapse. The CEA value for the advanced cancer group was 269.8 ± 1053.6; the CEA value for the non-advanced cancer group was 219.6 ± 719.3; and the P-value for both groups was 0.434. Anesthesia time was 341.9 ± 120.6 in the advanced cancer group, 326.2 ± 122.1 in the non-advanced cancer group and 0.050 in the two groups. ASA scores for the advanced cancer group and the non-advanced cancer group were different, and this result was statistically significant (P < 0.001). Similarly, there were significant differences in chemoradiotherapy between the advanced cancer group and the non-advanced cancer group, with p < 0.001 (See Table 1).

**Table 1:** Baseline data.

Progress	No	Yes	P-value*
N	221	778	
AGE (years)	68.9 ± 12.7	64.1 ± 13.8	<0.001
CEA	219.6 ± 719.3	269.8 ± 1053.6	0.434
LOGCEA	1.3 ± 0.9	1.4 ± 0.9	0.410
ANESTIME(min)	326.2 ± 122.1	341.9 ± 120.6	0.050
GENDER			0.924
Male	136 (61.5%)	476 (61.2%)	
Female	85 (38.5%)	302 (38.8%)	
ASA			<0.001
1	7 (3.2%)	46 (5.9%)	
2	113 (51.1%)	446 (57.3%)	
3	89 (40.3%)	277 (35.6%)	
4	11 (5.0%)	9 (1.2%)	
5	1 (0.5%)	0 (0.0%)	
DM			0.179
No	169 (76.5%)	627 (80.6%)	
Yes	52 (23.5%)	151 (19.4%)	
CAD			0.541
No	203 (91.9%)	724 (93.1%)	
Yes	18 (8.1%)	54 (6.9%)	
HF			0.456
No	209 (94.6%)	746 (95.9%)	
Yes	12 (5.4%)	32 (4.1%)	
CVA			0.076
No	203 (91.9%)	739 (95.0%)	
Yes	18 (8.1%)	39 (5.0%)	
CKD			0.227
No	185 (83.7%)	676 (86.9%)	
Yes	36 (16.3%)	102 (13.1%)	
LAPAROSCOPIC			1.000
No	213 (96.4%)	748 (96.1%)	
Yes	8 (3.6%)	30 (3.9%)	
EA			0.472
No	188 (85.1%)	646 (83.0%)	
Yes	33 (14.9%)	132 (17.0%)	
AJCC			0.105
No	134 (60.6%)	424 (54.5%)	
Yes	87 (39.4%)	354 (45.5%)	
LIVER_ONLY			0.259
No	132 (59.7%)	497 (63.9%)	
Yes	89 (40.3%)	281 (36.1%)	
CT			<0.001
No	78 (35.3%)	32 (4.1%)	
Yes	143 (64.7%)	746 (95.9%)	
RT			<0.001
No	213 (96.4%)	676 (86.9%)	
Yes	8 (3.6%)	102 (13.1%)	
NACTRT			0.081
No	195 (88.2%)	649 (83.4%)	
Yes	26 (11.8%)	129 (16.6%)	

Abbreviations: ASA physical status: American Society of Anesthesiologists physical status; CEA: carcinoembryonic antigen; CT: chemotherapy; RT: radiotherapy; CKD: Chronic kidney disease; CHF: Heart failure; CAD: Coronary arterial disease.

Note: The percentage of CEA AND LogCEA missing values was 0.099. The remaining variables have no missing values.

**Figure 1:**
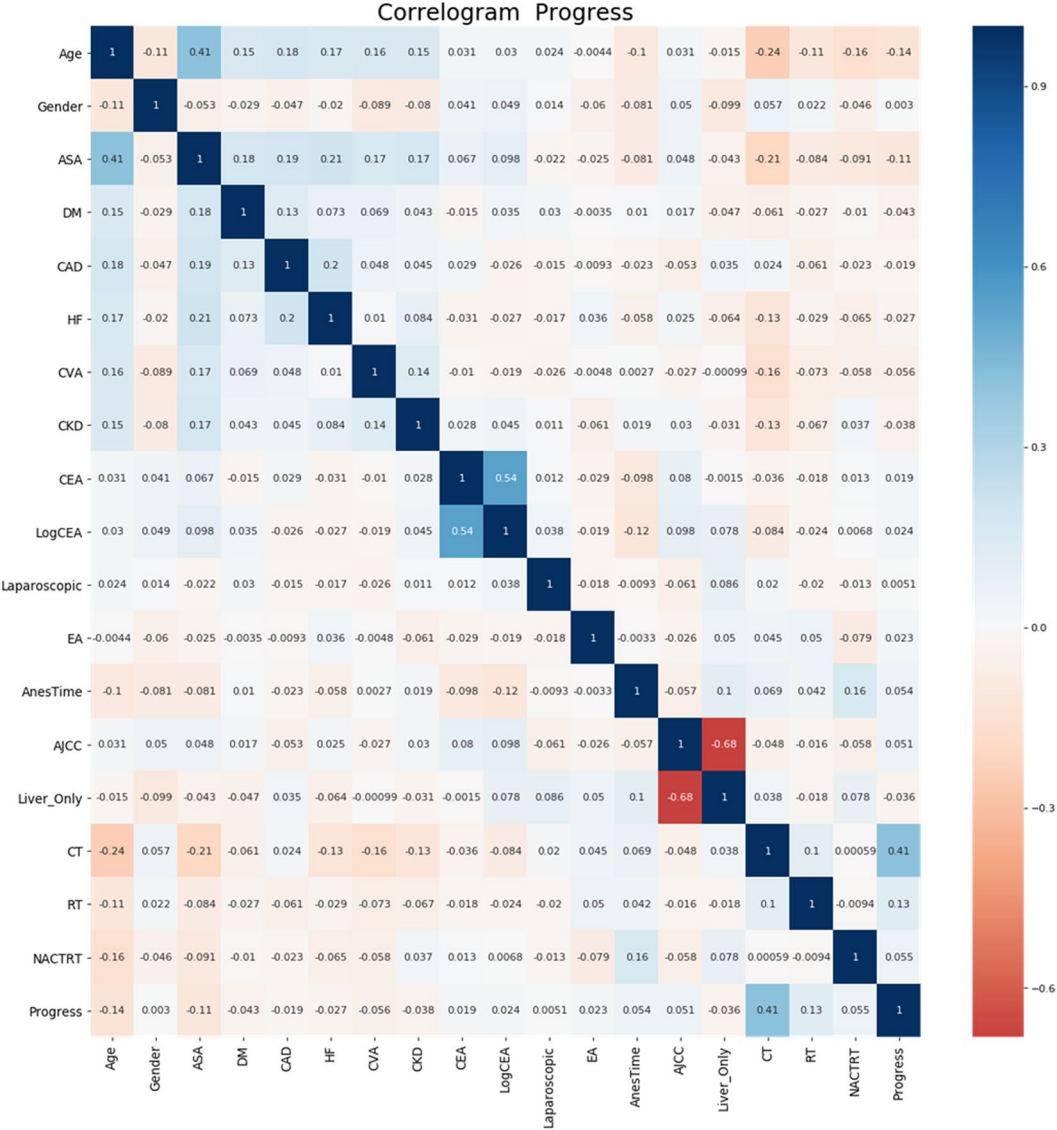
Correlation Analysis of Various Factors ASA physical status: American Society of Anesthesiologists physical status; CEA: carcinoembryonic antigen; CT: chemotherapy; RT: radiotherapy; CKD: Chronic kidney disease; CHF: Heart failure; CAD: Coronary arterial disease.

**Figure 2:**
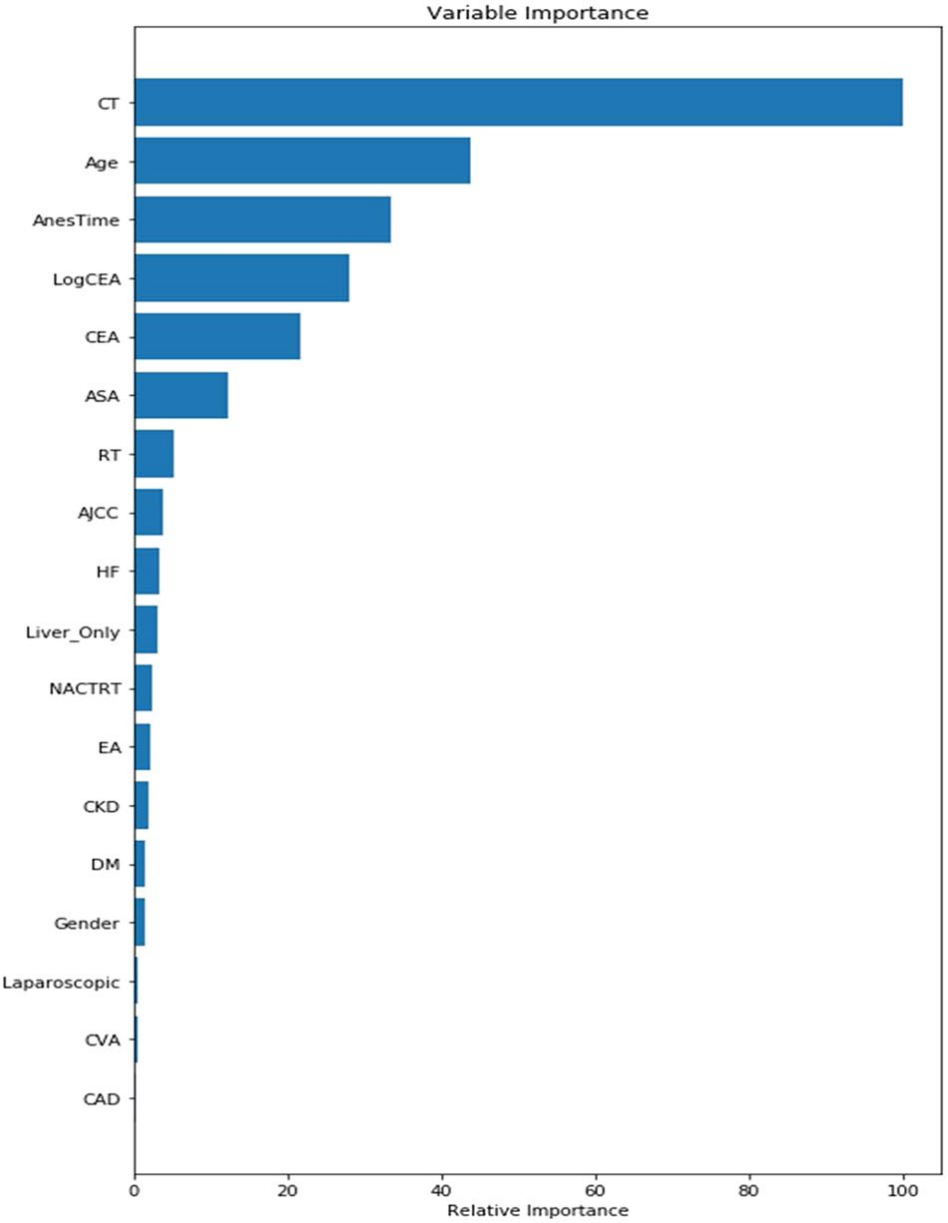
Variable importance of features included in machine learning GradientBoosting’s algorithm for prediction of Recurrence of colorectal cancer after tumor resection. Abbreviations: ASA physical status: American Society of Anesthesiologists physical status; CEA: carcinoembryonic antigen; CT: chemotherapy; RT: radiotherapy; CKD: Chronic kidney disease; CHF: Heart failure; CAD: Coronary arterial disease.

Figure 1 shows the correlation between the variables. It shows that age is negatively correlated with ASA and cancer progression. Chemotherapy and CEA are both positively correlated with cancer progression. Anesthetic time is also weakly positively correlated with cancer progression. Additionally, there is a weak negative correlation between anesthesia time and age and CEA. Figure 2 shows the importance of each covariate in GradientBoosting’s final model. The five most influential covariates are observable: chemotherapy, age, LogCEA, CEA and anesthesia time.

**Figure 3:**
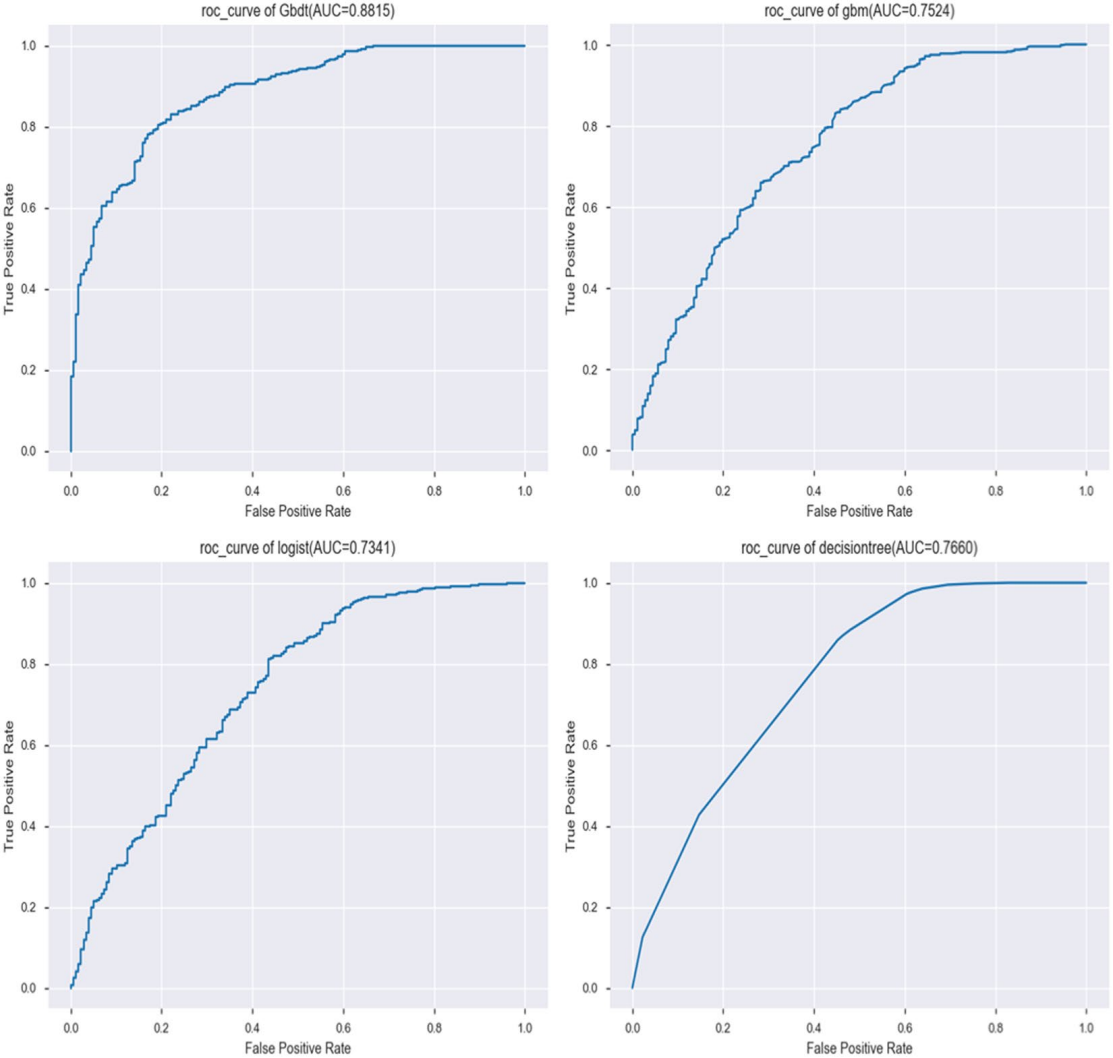
Machine learning algorithm for prediction of Recurrence of colorectal cancer after tumor resection in trainning group (Contains four machine learning algorithms, such as: logical regression, decision tree, GradientBoosting and lightGBM).

The four machine learning algorithms are compared in Table 2 and Fig. 3. The results for training group were: logistic regression model (AUC value = 0.734, accuracy = 0.828, precision = 0.842, recall = 0.958, F1 _ score = 0.896); Decision tree model (AUC value = 0.766, accuracy = 0.847, precision = 0.844, recall = 0.986, F1 _ score = 0.909); GradientBoosting model (AUC value = 0.881, accuracy = 0.851, precision = 0.841, recall = 0.997, F1_score = 0.912) and gbm model (AUC value = 0.752, accuracy = 0.825, precision = 0.841, recall = 0.955, F1 _ score = 0.895). This shows that the AUC value of the GradientBoosting model was the highest (0.881). The AUC value of the Logistic model was the lowest (0.734).

**Table 2:** Forecast Results of Training Group and Testing Group.

	Training Group		Testing Group							
	Accuracy	Precision	Recall	F1_score	AUC	Accuracy	Precision	Recall	F1_score	AUC
Logistic	0.827	0.842	0.958	0.896	0.734	0.830	0.828	0.987	0.901	0.692
DecisionTree	0.847	0.844	0.986	0.909	0.766	0.810	0.821	0.968	0.888	0.723
GradientBoosting	0.851	0.841	0.997	0.912	0.881	0.820	0.819	0.987	0.895	0.734
gbm	0.825	0.841	0.955	0.895	0.752	0.825	0.831	0.974	0.974	0.761

**Figure 4:**
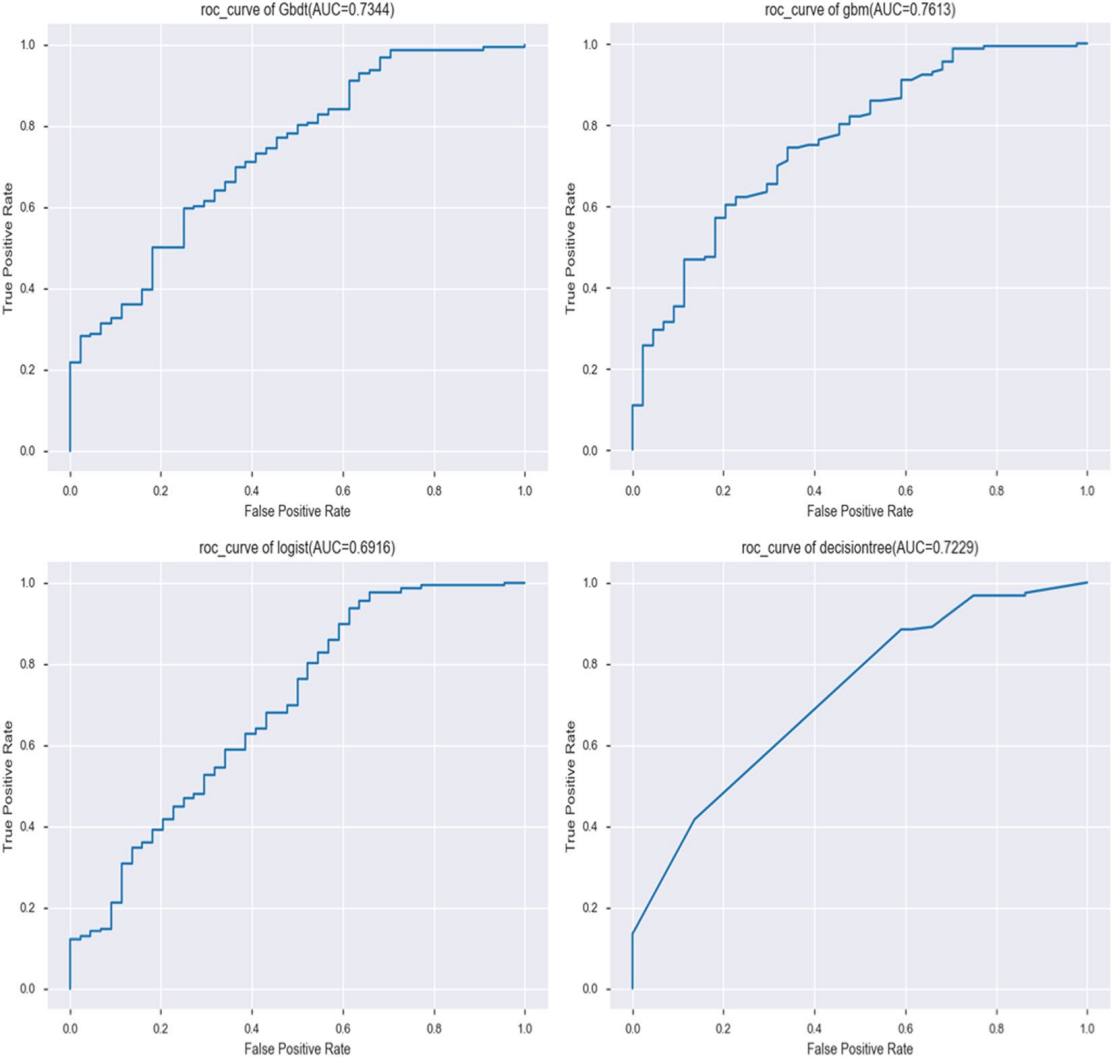
Machine learning algorithm for prediction of Recurrence of colorectal cancer after tumor resection in testing group (Contains four machine learning algorithms, such as: logical regression, decision tree, GradientBoosting and lightGBM).

The results of the test group were: logistic regression model (AUC value = 0.692, accuracy = 0.830, precision = 0.828, recall = 0.987, F1 _ score = 0.901); Decision tree model (AUC value = 0.723, accuracy = 0.810, precision = 0.821, recall = 0.968, F1 _ score = 0.888); GradientBoosting model (AUC value = 0.734, accuracy = 0.820, precision = 0.819, recall = 0.987, F1_score = 0.895) and gbm model (AUC Value = 0.761, accuracy = 0.825, precision = 0.831, recall = 0.974, F1 _ score = 0.974). This shows that the gbm model’s AUC value was the highest (0.761). The Logistic model’s AUC value was the lowest (0.692). The GradientBoosting model’s AUC value was 0.734, which can still predict cancer prognosis (See Table 2 and Fig. 4).

Different parameters influence the running results of various algorithms in machine learning. In this study, the final parameters for GradientBoosting were: learning _ rate = 0.01, n _ estimators = 100, min _ samples _ split = 10, min _ samples _ leaf = 1, subsample = 0.5, max _ depth = 5; The final parameters of gbm are: boosting _ type = ‘GBDT’, reg _ alpha = 0.001, reg _ lambda = 0.8, learning _ rate = 0.1, max _ depth = 1, n _ estimators = 100, objective = ‘binary’ (See Appendix Table 1).

## Discussion

Colorectal cancer has been on the rise in China in recent years. Due to neglect of early symptoms, some patients have already entered the advanced stages by the time they are admitted to the hospital. This increases the risk of death. According to the TNM staging criteria for colorectal cancer, tumors invading the serosal layer of the intestinal wall are considered stage T4. According to previous studies, although the short-term effect of radical surgery for T4 patients is ideal, the long-term effect is poor, and the recurrence and metastasis rates are high^17^. In this study, the progression rate for postoperative cancer in patients with stage IV colorectal cancer was as high as 77.9%. Our study compared four ML algorithms using real-world data and found that DecisionTree, GradientBoosting and gbm algorithms can better predict the postoperative cancer progression of patients with stage 4 colorectal cancer, in both training and testing groups. Furthermore, it was found that the five most influential covariates were chemotherapy, age, LogCEA, CEA and anesthesia time. These variables are correlated. For example, there is a significant positive correlation between chemotherapy and cancer progression. Anesthetic time is also weakly positively correlated with cancer progression.

Postoperative chemoradiotherapy is a standard adjuvant therapy for patients with T3-4 and/or lymph node-positive rectal cancer. Long-term postoperative radiotherapy can reduce local recurrence by 50% to 60%, compared to surgery alone^18^. Simultaneous addition of fluoropyrimidine chemotherapy and radiotherapy can further reduce systemic metastasis and local recurrence^19^. At present, the most controversial issue is whether postoperative radiotherapy is necessary for those with low risk of local recurrence, as indicated by the postsurgical pathology. An example of this would be patients with upper rectal cancer or who are staged as T1-2N1 or T3N0. Retrospective studies in a single institution have shown that some T3N0 patients may not require postoperative radiotherapy^20^. Furthermore, among patients with advanced cancer, early palliative care may optimize patient selection for chemotherapy reducing the use of high-intensity therapy by focusing on quality of life in accordance with patients’ performance, preferences and care goals^21^. Additionally, no clear linear pattern between adjuvant chemotherapy and better adjusted relative survival in colon cancer was observed^22^. These results did not indicate that radiotherapy and chemotherapy will benefit patients with stage IV colorectal cancer after surgery. This may only reflect that surgery can be applied to patients at later stages.

In recent years, the incidence and mortality of colorectal cancer have risen, and the age of onset has become younger^23^. Our study also showed that the age of the cancer progression group was younger than that of the non-progression group, but that age still accounted for a large weight of cancer progression.

Serum CEA is an acidic glycoprotein with human embryo antigen specificity. It is an important marker of digestive tract tumors. Serum tumor markers are common in tumor diagnosis. Many studies^24–26^ have evaluated the role of CEA, CA19-9 and CA50 in the diagnosis, prognosis and recurrence monitoring of colorectal cancer. Similarly, this study also showed that LogCEA is an important factor in the progression of stage IV colorectal cancer patients after surgery.

Surgical injury and anesthesia can cause a bodily stress response, affecting immune response and causing reversible immune function changes in the body. This study found that anesthesia time is an important weight for cancer progression. This may be related to changes in immune function among patients with perioperative cancer caused by anesthesia.

A follow-up study conducted by Bonjer *et al*.^27^ showed that the 3-year disease-free survival rates for patients after LS and OS surgery were 74.8% and 70.8%, respectively. The results obtained by COREAN^28^ were 79.2% and 72.5%, respectively. However, there was no significant difference between LS and OS in local recurrence, disease-free survival, or overall survival after RC. However, in this study, laparoscopic surgery was found to promote tumor progression in patients with stage IV colorectal cancer. This may be related to the application of CO_2_ pneumoperitoneum in laparoscopic surgery. This affects patients’ immune function, thereby increasing the risk of tumor metastasis and recurrence, thus influencing prognosis.

The incidence of colorectal cancer ranks third among the most common malignancies among men and second among women. It is the fourth leading cause of cancer-related mortality worldwide^23^. In this study, sex was also found to be a factor in the progression of postoperative patients with stage IV colorectal cancer.

The anatomical features of portal vein blood backflow determine whether the liver is the most common distant metastatic site of colorectal cancer. Hepatic metastases were found in 20% of patients when they were diagnosed with colorectal cancer. This makes it difficult to treat, and the prognosis is usually bleak. This is similar to the findings of the present study.

This retrospective and observational study has several limitations. Firstly, patients were not randomized, the comparisons between ML prediction and statistical prediction groups were not conducted, and clinical care was not standardized. Therefore, the effects of selection bias and unmeasured confounding variables could not be excluded. Secondly, due to data requisition limitations, data on total anesthesia requirements, perioperative analgesia and intraoperative chemotherapy for each patient (such as high-temperature intraperitoneal chemotherapy) were unavailable. Thirdly, different parameters for each ML algorithm may have resulted in different results.

## Conclusion

GradientBoosting and gbm are more likely to improve the accuracy of predicting the postoperative cancer progression of patients with stage IV colorectal cancer than are the other two ML algorithms. Furthermore, set algorithms are more effective than basic algorithms. The five most influential covariates in cancer progression after surgery for stage 4 colorectal cancer patients are chemotherapy, age, LogCEA, CEA and anesthesia time. Anesthetic time has a weak positive correlation with cancer progression. Additional multicenter clinical studies are needed in the future.

## Data Availability

Data are available at the BioStudies database (https://www.ebi.ac.uk/biostudies/studies/S-EPMC6054421), accession number: S-EPMC6054421.

## Appendix

**Table A1:** Functions, Packages, and Tuning Parameters in the anaconda Software Used for Each Machine Learning Algorithm.

Algorithm	Classifier	Package	Tuning Parameters
Logistic regression	LogisticRegression	Sklearn 0.19.1 (from sklearn.linear_model import LogisticRegression)	penalty = ‘l2’, tol = 0.0001, C = 0.7, fit_intercept = True,intercept_scaling = 1, class_weight = None, max_iter = 100, multi_class = ‘ovr’, verbose = 0,warm_start = False, n_jobs = −1
DecisionTree	DecisionTreeClassifier	Sklearn 0.19.1 (from sklearn.tree import DecisionTreeClassifier)	criterion = ‘gini’, splitter = ‘best’, max_depth = 7, min_samples_split = 20, min_samples_leaf = 1, min_weight_fraction_leaf = 0.0, max_features = None, random_state = None, max_leaf_nodes = None, min_impurity_decrease = 0.0, min_impurity_split = None, class_weight = None, presort = False
GradientBoosting	GradientBoostingClassifier	Sklearn 0.19.1 (from sklearn.ensemble import GradientBoostingClassifier)	learning_rate = 0.01, n_estimators = 100, min_samples_split = 10, min_samples_leaf = 1, subsample = 0.5, max_depth = 5
gbm	lgb.LGBMClassifier	lightgbm 2.2.0	boosting_type = ‘gbdt’, reg_alpha = 0.001, reg_lambda = 0.8, learning_rate = 0.1, max_depth = 1, n_estimators = 100, objective = ‘binary'
